# Supplementary material for: Targeting HER2-Positive Solid Tumors with CAR NK Cells: CD44 Expression Is a Critical Modulator of HER2-Specific CAR NK Cell Efficacy
Source: Cancers (Basel). 2025 Feb 21;17(5):731. doi: 10.3390/cancers17050731 (PMC11898473; doi:10.3390/cancers17050731)
Supplement: Supplementary file 1 [file cancers-17-00731-s001.zip › cancers-3478210-supplementary.pdf]

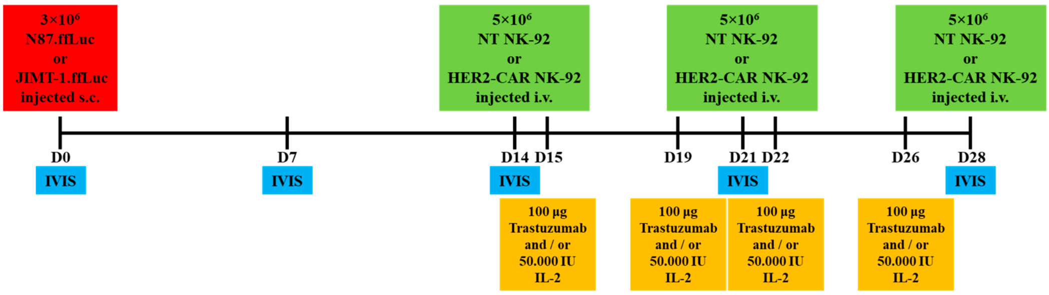

Figure S1. Schedule and applied treatments of the in vivo mouse experiment. A total of 25 female NOD.Cg-Prkdcscid/Il2rgtm1Wjl/SzJ mice received 3 × 10⁶ N87.ffLuc or JIMT-1.ffLuc cells subcutaneously in 100µl PBS and 100µl Matrigel (red box). Effector cell treated mice received on day 14, and then biweekly (green box), an i.v. dose of 5 × 10⁶ NT NK-92 cells or HER2-CAR NK cells. NT NK-92 plus trastuzuma-treated animals received 100 µg trastuzumab in 100 µl PBS i.p. twice a week during the experiment, starting from day 15 (orange box). All treated animals received 50000 IU IL-2 in 100 µl PBS i.p. twice a week during the experiment, starting from day 15 (orange box). Tumor growth was followed by weekly bioluminescence imaging (blue box).

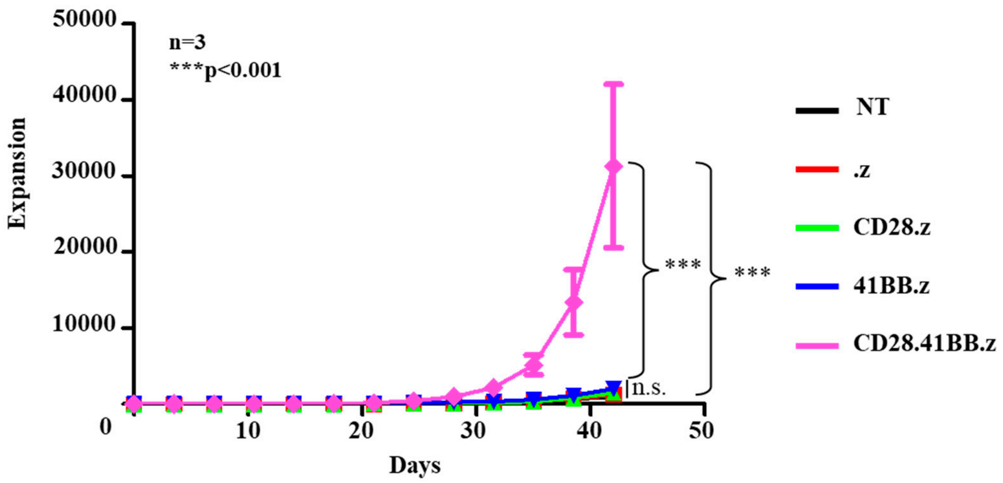

Figure S2. Quantitative cell proliferation data from culture day 0 to 42. 1 × 10⁵ HER2-CAR NK and NT NK cells were plated in duplicates in the presence of 400 IU/ml interleukin-2. Every 3.5 days, the effector cell number was determined by flow cytometry, and then the initial effector cell quantity was placed onto new plates in conditions identical to the beginning of the experiment. The expansion rate was calculated as the ratio of cells at the end to at the beginning of the 3.5-day period. Histograms represent the mean ± SD (n = 3; assay in du-plicates); ***p<0.001.

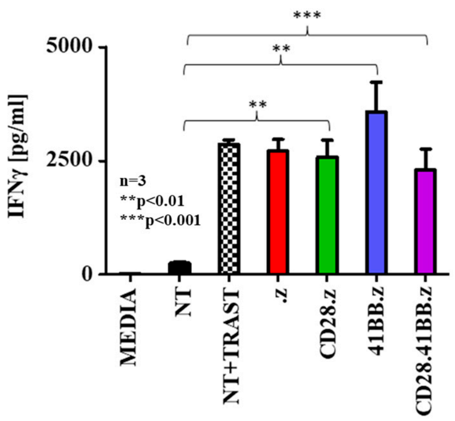

Figure S3. HER2-specific CAR-NK cells successfully recognized immobilized HER2 target: 1 × 10[5] HER2-CAR NK or NT NK cells ± 10 μg/ml trastuzumab were incubated on HER2-Fc protein-coated plates. After 24 h, IFNγ was determined in the culture supernatant by ELISA (n = 3, assay performed in duplicates). Histograms show mean ± SEM; **p<0.01; ***p<0.001.

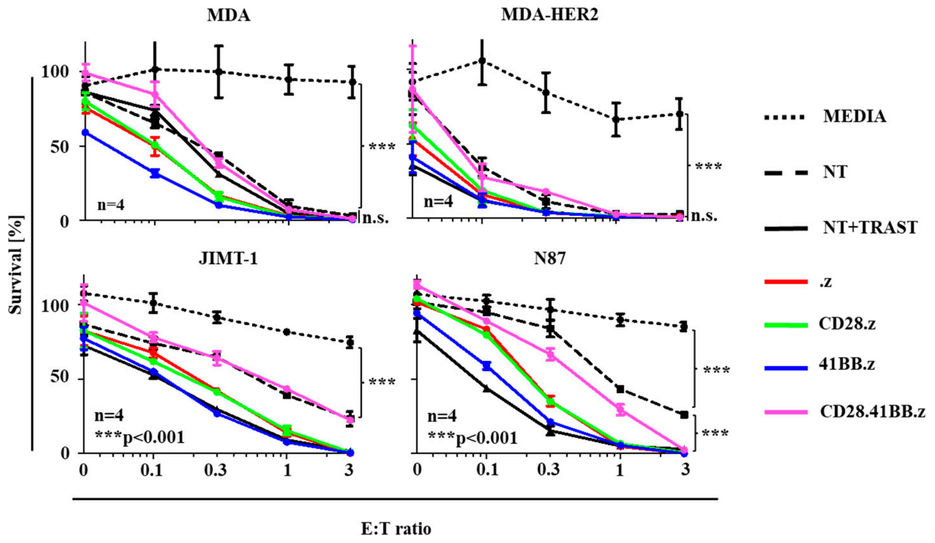

Figure S4. Firefly-Luciferase-based cytotoxicity assay. HER2-CAR NK or NT NK cells ± 10 μg/ml trastuzumab against 3 × 10[4] MDA-HER2, JIMT-1 or N87 (HER2+)or MDA (HER2-) target cells in at 1 – 0.3 - 0.1 - 0.03 – 0.01 – 0.003:1 NK cell to tumor cell ratio (marked as E:T ratio in the figure). Cell culture media was supplemented with 400 IU/ml IL-2. (n = 3; assay was performed in duplicates) Histograms show mean ± SEM; ***p<0.001. Non-treated NK-92 cells were compared to media control and additionally, the NT NK-92 cells were compared to the .z group at the effector-to-target ratio of 3:1.
